# Cervical Myelopathy Caused by Engorged Epidural Cervical Plexus Associated with Iatrogenic Intracranial Hypotension

**DOI:** 10.5334/jbsr.2797

**Published:** 2022-09-21

**Authors:** Bjorn Valgaeren, Elyn Van Snick, Geert Heirwegh

**Affiliations:** 1 General Hospital Damiaan Ostend, BE

**Keywords:** Intracranial Hypotension, Iatrogenic Overdrainage, Brain MRI, Cervical Spine MRI, Epidural Venous Plexus, Myelopathy, Spinal Cord Compression, Arachnoid Cyst

## Abstract

**Teaching Point:** Iatrogenic overdrainage of cerebrospinal fluid may cause intracranial hypotension with secondary engorgement of the epidural venous plexus, resulting in potentially reversible compression radiculopathy or myelopathy.

## Case History

A 47-year-old female with clinical history of cerebral arachnoid cyst shunting presented herself at the neurosurgeon’s consultation with a slowly progressive headache, new onset of neck pain, and right-sided brachialgia. The arachnoid cyst volume was stable on follow-up brain computed tomography (CT) scan. Radiography showed no shunt disconnection and showed normal position of the shunt valve.

**Figure 1:**
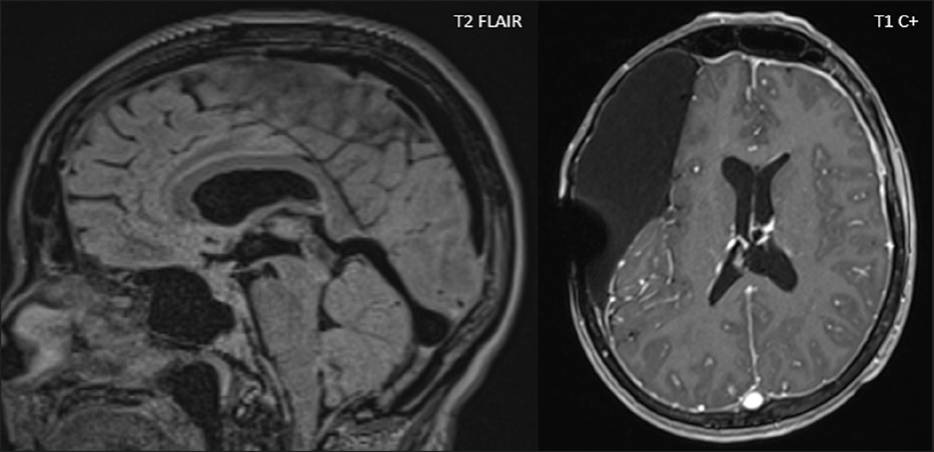


Magnetic resonance imaging (MRI) of the brain showed no significant changes in the arachnoid cyst. However, diffuse pachymeningeal enhancement with enlarged venous sinuses, reduced pontomesencephalic angle, reduced mamillopontine distance, enlarged pituitary gland, ‘droopy penis sign’, and ‘venous distension sign’ were seen (Figure 1), compatible with intracranial hypotension.

**Figure 2:**
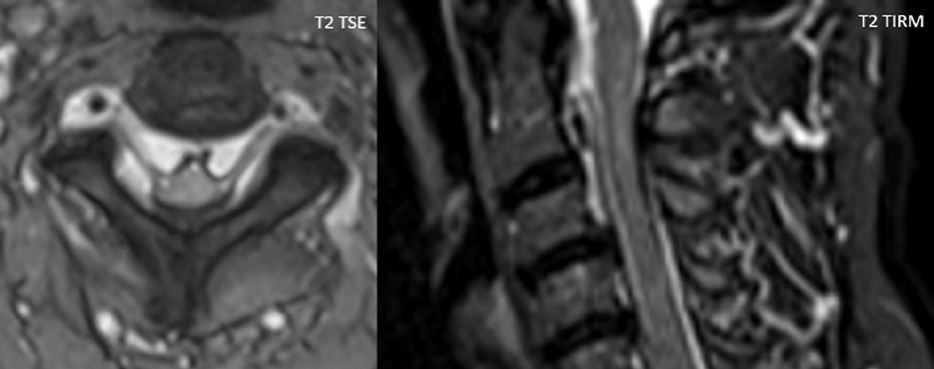


Cervical spine MRI showed engorged epidural cervical plexus with obliteration of the intervertebral foramina on multiple levels and compression of the spinal cord with subtle T2-hyperintense changes in the spinal cord at level C2-C3 (Figure 2), compatible with myelopathy.

Diagnosis of iatrogenic overdrainage of the arachnoid cyst with secondary epidural venous plexus engorgement and compression myelopathy was suggested. Overdrainage correction by shunt valve adjustments resulted in slow resolution of the engorged epidural venous plexus, confirming the diagnosis.

## Comment

Major imaging signs of intracranial hypotension on brain MRI include diffuse pachymeningeal enhancement, subdural fluid collections, dural venous enlargement, and evidence of brain descent [1]. Meningeal enhancement is believed to be caused by accumulation of gadolinium-based contrast in the engorged dural veins and dural interstitium [1]. Subdural collections can be seen, mostly bilateral and without significant mass effect [1].

Cervical spine MRI after intravenous gadolinium contrast administration reveals diffuse enhancement of the epidural venous plexus, which is symmetrically located in the anterolateral aspect of the cervical spinal canal, sparing the posterior aspect and midline. Flow artefacts can occasionally be seen [1]. T2-hyperintense alterations can be seen in the spinal cord in case of compression myelopathy.

Intracranial hypotension is believed to be a result of low cerebrospinal fluid (CSF) volume due to rupture of a spinal arachnoid membrane, which allows CSF leakage into the subdural or epidural space. Leakage can occur spontaneously, after (minor) trauma or due to iatrogenic procedures like lumbar punctures or overdraining spinal or ventricular shunts [1].

Because CSF volume and intracranial blood volume are inversely correlated according to the Monro-Kellie rule, intracranial blood volume rises to maintain intracranial pressure. Subsequently, the spinal epidural venous plexus enlarges and may compress nerve roots or spinal cord potentially resulting in radiculopathy or myelopathy [1].

Treatment is based on correction of the CSF leakage. Conservative treatment is preferred; however, surgery might be necessary. CSF shunts can be adjusted [1].
